# Central Multifocal Choroiditis: Platelet Granularity as a Potential Marker for Treatment With Steroid-Sparing Immunomodulatory Therapy

**DOI:** 10.3389/fopht.2021.784848

**Published:** 2021-11-25

**Authors:** Evianne L. de Groot, Jeannette Ossewaarde-van Norel, Imo E. Hoefer, Saskia Haitjema, Joke H. de Boer, Jonas J. W. Kuiper

**Affiliations:** ^1^ Department of Ophthalmology, University Medical Center Utrecht, Utrecht University, Utrecht, Netherlands; ^2^ Central Diagnostic Laboratory, University Medical Center Utrecht, Utrecht University, Utrecht, Netherlands; ^3^ Center for Translational Immunology, University Medical Center Utrecht, Utrecht University, Utrecht, Netherlands

**Keywords:** multifocal choroiditis, MFC, PIC, punctate inner choroidopathy, IMT, corticosteroid-sparing immunomodulatory therapy, posterior uveitis, blood cell composition

## Abstract

**Purpose:**

We aimed to evaluate the blood cell composition in patients with central multifocal choroiditis (cMFC), a rare form of posterior uveitis predominantly affecting young myopic women.

**Methods:**

In this retrospective observational case-control study, a 104-parameter automated hematocytometry was conducted by the Cell-Dyn Sapphire hematology analyzer for 122 cases and 364 age- and sex-matched controls. Cox proportional regression analysis was used to assess the relation between the blood cell composition and the time between disease onset (first visit) and the start of systemic corticosteroid-sparing immunomodulatory therapy (IMT).

**Results:**

At a false discovery rate of 5% (P_adj_), we identified a decrease of blood monocytes in cases with cMFC, which could be attributed to disease activity. Cox proportional hazard analysis including age and sex revealed that increased platelet granularity (measured by mean intermediate angle scatter) was an independent risk factor for treatment with IMT (hazard ratio = 2.3 [95% confidence interval = 1.28 - 4.14], P_adj_ = 0.049). The time between the first presentation and the start of IMT was 0.3 years in the group with an increased platelet granularity and 3.4 years in the group without increased platelet granularity.

**Conclusions:**

Patients with cMFC demonstrated a decrease in blood monocytes. Moreover, platelet granularity could potentially be used as a marker for treatment with IMT.

## Introduction

Central multifocal choroiditis (cMFC) is a group of inflammatory disorders of the choroid in the macular area of eyes. Predominantly young myopic Caucasian women are affected and the disease is often sight-threatening (1, 2). The clinical hallmark of white-yellowish lesions classifies these conditions among the white dot syndromes, and typically comprises several subtypes including *punctate inner choroidopathy (PIC)* (3)*, multifocal choroiditis (MFC)* (4)*, relentless placoid chorioretinitis (RPC)* (5)*, persistent placoid maculopathy (PPM)* (6) and *serpiginous choroiditis (SC)* (7). CMFC is further characterized by hypofluorescent areas on indocyanine green angiography (ICGA) which in the active phase of the disease increase in size and have blurred margins. Moreover, it is typified by the absence of papillitis or vasculitis, which are often seen in other types of non-infectious uveitis. The etiology of cMFC is poorly understood, but the beneficial effect of systemic corticosteroids or corticosteroid-sparing immunomodulatory therapy (IMT) (e.g., methotrexate, adalimumab) on disease activity visualized with ICGA supports that inflammatory mechanisms drive the pathophysiology of cMFC (8–11). In addition, small genetic studies indicated that susceptibility to cMFC is linked to immune genes including *IL10* and *TNF* loci, complement factor H (*CFH*) and increased prevalence of the Human Leukocyte Antigen DR2 (12–15). Immune profiling studies of cMFC are currently lacking. In this study, we aimed to compare the peripheral blood cell composition of patients with cMFC to control subjects. Secondly, we aimed to explore the relationship between the blood cell composition and disease severity using treatment with IMT as surrogate marker for disease severity.

## Materials and Methods

This observational case-control study was conducted in accordance with the Declaration of Helsinki including all its amendments. All patients were ≥18 years old and provided written informed consent to use their medical data for research purposes. The institutional review board of the University Medical Centre (UMC) of Utrecht approved this study.

### Study Participants

Patients were diagnosed with cMFC in case they presented with chorioretinal scars in the posterior pole and without papillitis and vasculitis. Patients were subdivided in different subtypes based on the classification criteria of the Standardization of Uveitis Nomenclature working group for PIC, MFC and SC (3, 4, 7). In case of overlapping criteria for different subtypes, this was resolved with discussion. Moreover, patients with RPC and PPM were diagnosed based on typical appearance on multimodal imaging (5, 6). On indication, other causes of inflammatory eye diseases were ruled out by diagnostic work-up for uveitis, including soluble interleukine-2 receptor, angiotensin converting enzyme, QuantiFERON-TB, HLA-B27 and HLA-A29 typing and X-ray of the chest.

To enable comparison of cMFC blood cell composition with a reference, for every cMFC patient, three control subjects were extracted from the UPOD database. These control subjects were healthy individuals that underwent a periodic occupational health examination and were matched by sex and age with maximum age deviation of four years.

### Blood Cell Composition Analysis

Automated blood cell composition analyses were performed with the Abbott Cell-Dyn Sapphire (Abbott Diagnostics, Santa Clara, CA, USA) hematology analyzer and data for 104 parameters were analyzed and stored by the Utrecht Patient Oriented Database (UPOD) of the University Medical Centre Utrecht. The Abbott Cell-Dyn Sapphire automated blood cell analyzer uses five optical scatter signals measuring cell size [0 degrees scatter, axial light loss (ALL)], cell complexity and granularity [7 degrees scatter, intermediate angle scatter (IAS)], nuclear lobularity [90 degrees scatter, polarized side scatter (PSS)], depolarization [90 degrees depolarized side scatter (DSS)] and viability [red fluorescence 90 degrees (FL3), 630 ± 30 nm]. The structure and content of UPOD have been described in more detail elsewhere (16). The blood cell analyzer is equipped with an integrated 488-nm blue diode laser and uses spectrophotometry, electrical impedance, laser light scattering (multi angle polarized scatter separation), and 3-color fluorescent technologies to measure morphological parameters of leukocytes, red blood cells, and platelets for classification and enumeration (17, 18). Details on the blood cell parameters measured by the Cell-Dyn Sapphire analyzer is provided in **Supplementary Table S1**. UPOD stores blood cell data for >3 million samples from the University Medical Centre Utrecht. Data on disease activity and treatment were extracted from the electronic patient records. If blood cell composition data was available for multiple visits, preferably a sample collected during active disease without systemic immunomodulatory therapy (n=76) was used. If not available consecutively we used a sample collected during inactive disease without systemic immunomodulatory therapy (n=18), during active disease with systemic immunomodulatory therapy (n=23) and during inactive disease with systemic immunomodulatory therapy (n=5) (**Table 1**).

**Table 1:** Patient characteristics: disease activity and systemic immunomodulatory therapy.

Total of 122 patients	Active disease	Inactive disease
Number of patients without systemic immunomodulatory therapy	76	18
Number of patients treated with systemic corticosteroids	13	3
Number of patients treated with IMT	4	1
Number of patients treated with systemic corticosteroids + IMT	6	1

In the patient group (n=122), blood samples for blood cell composition analysis were selected based on the presence of disease activity and treatment with systemic immunomodulatory therapy (systemic corticosteroids or IMT).

IMT, corticosteroid-sparing immunomodulatory therapy.

### Statistical Framework

Data analyses were performed in RStudio version 1.2.5001 (RStudio Team, Boston, USA) and R version 4.0.0 (R Foundation for Statistical Computing, Austria). Principal component analysis (PCA) was performed in 111 patients and 323 control subjects with complete data using the *factoextra* R package (19) after scaling of the data. Group differences were tested using a likelihood ratio test (LRT) with a false discovery rate (FDR) of 5% and corrected for disease activity and treatment with systemic corticosteroids respectively (added as a covariate to the linear models). For survival analysis, feature selection was conducted by filtering for parameters that were different (LRT, age and sex adjusted *P_LRT_* <0.05) between cases with early start of corticosteroid-sparing immunomodulatory therapy (IMT within 6 months after the first presentation in the UMC Utrecht) and cases starting IMT after 6 months after the first presentation in the UMC Utrecht. An optimal cut off point was calculated based on the maximum log-rank statistic using the *surv_cutpoint()* function of the *survminer* R package (20) with a minimal proportion of observations per group of 0.3. This cut off point was used for a survival analysis and the hazard ratios of these parameters were explored using a cox proportional hazards model with age and sex as covariates using the R package *survival* (21).

## Results

### Study Population

Retrospective search by UPOD revealed available blood cell composition data for 122 cMFC patients measured between May 2005 and April 2020. The median age (range) at time of blood sampling of the 122 patients was 42 (16-80) years and 104 patients (85%) were female. The control group consisted of 364 individuals, of which 311 (85%) females, and with a median age (range) of 42 (17-83). Thirty-four (28%) patients were diagnosed with punctate inner choroidopathy (PIC), 71 (58%) patients with multifocal choroiditis (MFC), 2 (2%) patients with persistent placoid maculopathy (PPM), 4 (3%) patients with serpiginous choroiditis (SC) and 11 (9%) patients with relentless placoid chorioretinitis (RPC).

### Decreased Blood Monocyte Count in Patients With cMFC

**Figure 1:**
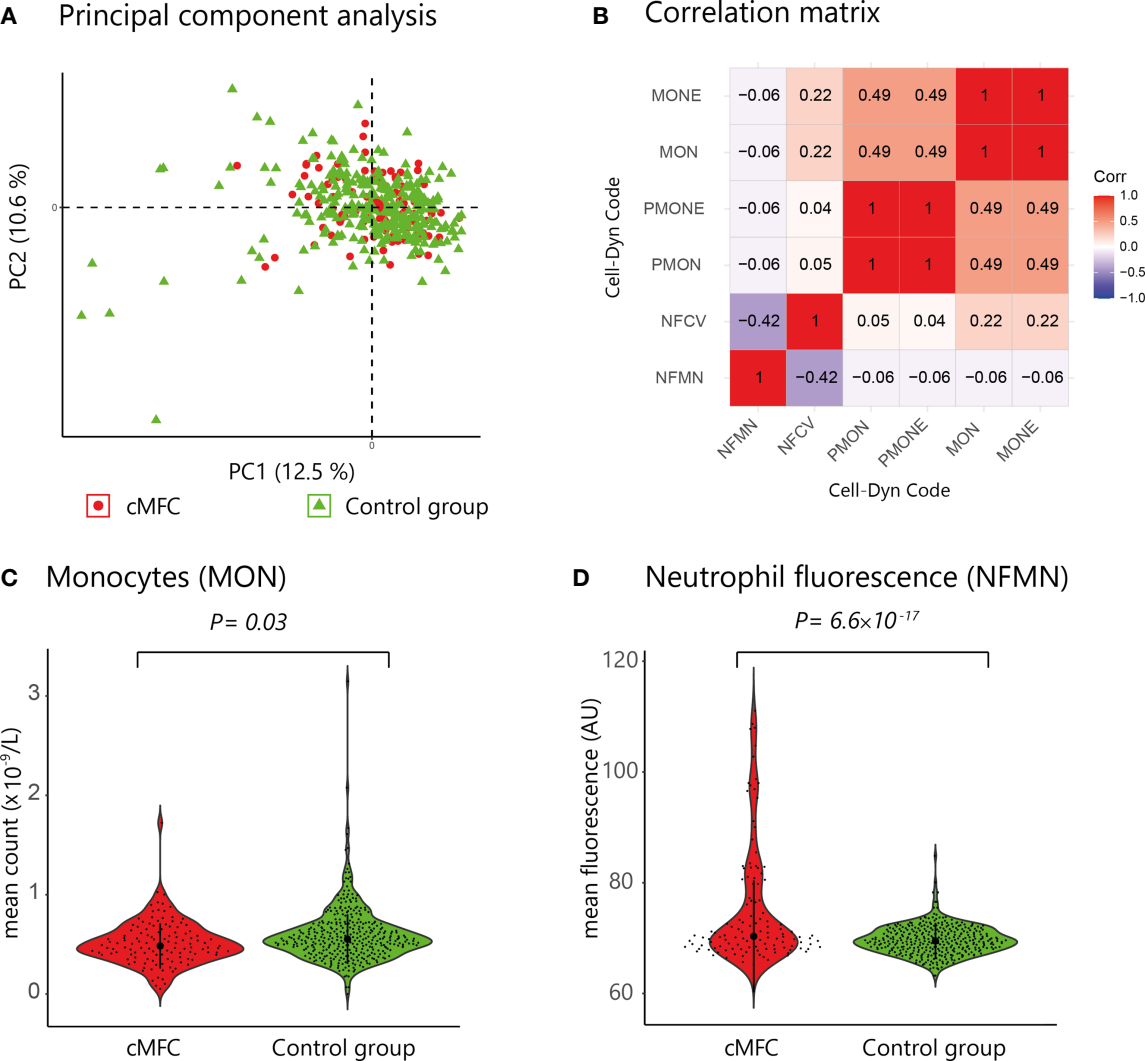
**(A)** Principal component analysis of blood cell count data from patients (red dots) and controls subjects (green triangles). **(B)** Correlation matrix of the six parameters associated with cMFC (Likelihood ratio test, Padj<0.05) **(C, D)** Violin plots of the mean monocyte count (Cell-Dyn code MON) and the neutrophil fluorescence (NFMN) for cMFC cases and controls. NFMN, mean FL3-fluorescence of neutrophil granulocytes; NFCV, the coefficient of variance as percentage of the mean of the FL3-fluorescence of neutrophil granulocytes; MON, number of monocytes; MONE, absolute number of monocytes without blasts; PMON, monocytes as percentage of all leukocytes; PMONE, the number of monocytes without blasts as percentage of all leukocytes; PC, principal component.

Principal component analysis revealed no global differences between the cMFC and reference population (**Figure 1A**), indicating that the blood profiles were largely comparable. At a false discovery rate (FDR) of 5%, we detected differences for 6 neutrophil and monocyte parameters (**Table 2**, and **Figures 1B**–**D**); specifically, the monocyte count and percentage of monocytes were decreased in cMFC patients (**Table 2**). Adjusting for disease activity (i.e., added as a covariate in the linear models) mitigated the signal for monocytes (*P_adj_* > 0.05). Adjusting for treatment with systemic corticosteroids did not change the signal for monocytes, indicating that the decrease of monocytes could be primarily attributed to disease activity in patients.

**Table 2:** Differences in blood cell composition parameters between patients with cMFC and healthy controls.

Description	Cell-Dyn Code	cMFC (n = 122) median (IQR)	Controls (n = 364) median (IQR)	*P_adj_*	*P_adj_ - corrected for disease activity*	*P_adj_ - corrected for systemic corticosteroids*
Neutrophil fluorescence (AU)	NFMN	70.3 (68.4 - 77.9)	69.5 (68.0 - 71.3)	6.6×10^-17^	0.04	3.5×10^-16^
Neutrophil fluorescence (AU)	NFCV	7.6 (6.2 - 9.1)	8.2 (6.9 - 9.2)	0.03	0.86	5.9×10^-3^
Blood monocyte count (x 10^9^/L)	MON	0.48 (0.40 - 0.62)	0.56 (0.44 - 0.69)	0.03	0.87	5.9×10^-3^
Blood monocyte count (without blasts) (x 10^9^/L)	MONE	0.48 (0.39 - 0.62)	0.56 (0.44 - 0.69)	0.02	0.87	5.9×10^-3^
Blood monocyte %	PMON	6.52 (5.35 - 7.88)	7.33 (6.08 - 8.95)	4.3×10^-3^	0.87	0.06
Blood monocyte % (without blasts)	PMONE	6.52 (5.33 - 7.88)	7.33 (6.08 - 8.95)	4.1×10^-3^	0.87	0.05

The adjusted P-values (false discovery rate of 5% from likelihood ratio test) (P_adj_) are indicated with and without including disease activity or systemic corticosteroids as covariate in the linear model.

cMFC, central multifocal choroiditis; IQR, interquartile range; AU, arbitrary units; NFMN, mean FL3-fluorescence of neutrophil granulocytes; NFCV, the coefficient of variance as percentage of the mean of the FL3-fluorescence of neutrophil granulocytes; MON, number of monocytes; MONE, absolute number of monocytes without blasts; PMON, monocytes as percentage of all leukocytes; PMONE, the number of monocytes without blasts as percentage of all leukocytes.

We also detected enhanced FL3 fluorescence of blood neutrophils (Cell-Dyn parameter; *NFMN*) in cMFC patients (**Table 2**). After correcting for disease activity, the increased fluorescence of neutrophils (NFMN) remained significant (*P_adj_* = 0.04) (**Table 2**). Since enhanced fluorescence of neutrophils is unexpected [only reported in patients using certain anti-psychotic drugs (18)], we used available clinical parameters to infer possible confounders. To this end, we stratified cases according to the mean fluorescence of neutrophils of all patients (cut-off NFMN parameter = 75.1). Comparison of cases with relatively high and low fluorescence of neutrophils revealed comparable distributions for age and sex (**Table 3**). In contrast, 94% of cases with increased fluorescence in neutrophils (NFMN>75.1) were subjected to fluorescein and indocyanine green angiography within 8 hours prior to blood sampling compared to 8% of cases without increased fluorescence of neutrophils (χ^2^; *P =* 6.9 ×10^-19^) (**Table 3**). This suggests that the increased fluorescence of blood neutrophils in cMFC was most likely directly related to exposure to fluorescent dyes for ocular imaging just prior to blood cell measurement.

**Table 3:** Clinical characteristics associated with neutrophil fluorescence.

	Low NFMN (n = 85)	High NFMN (n = 35)	*P-*value
Mean age^*^	42.7	41.0	0.49
Female/cases (%)^†^	70/85 (82.4%)	32/35 (91.4%)	0.33
FA-ICGA (%)^†,‡^	7/85 (8.2%)	33/35 (94.3%)	6.9 ×10^-19^

The patients with available data on neutrophil fluorescence (NFMN) (n=110) were divided in a group with a low NFMN (n=85) and high NFMN (n=35) using the mean of all patients of 75.1 arbitrary units as a cut-off point.

NFMN, mean FL3-fluorescence of neutrophil granulocytes; FA-ICGA, fluorescein and indocyanine green angiography.

^*^Student’s t-test.

^†^Chi-square (χ^2^) test.

^‡^The FA-ICGA was performed within 8 hours prior to blood sampling.

### Platelet Granularity Is Associated With Systemic Corticosteroid-Sparing Immunomodulatory Treatment

**Figure 2:**
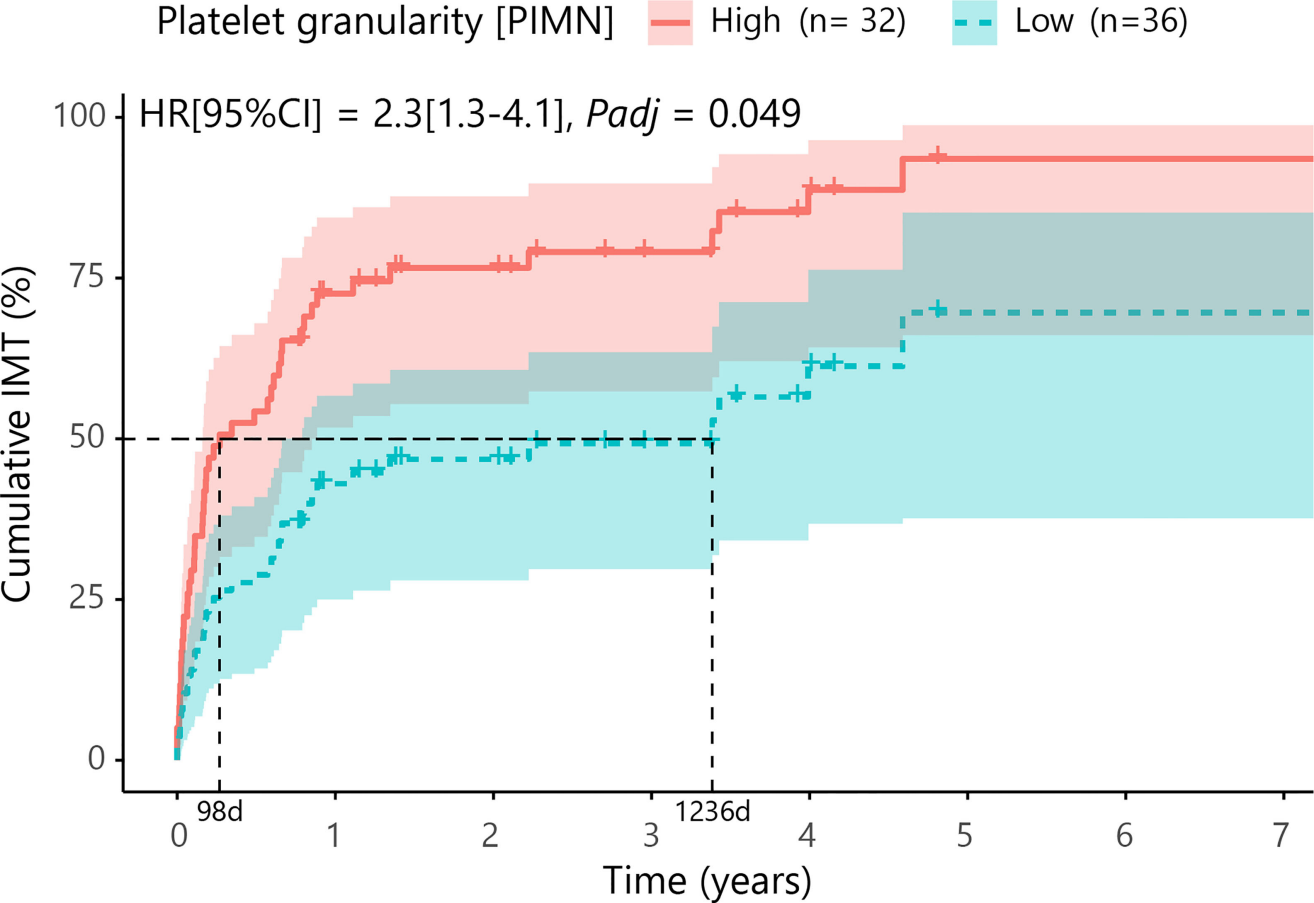
Cumulative incidence curve (Cox proportional hazards model corrected for age and sex with false discovery rate correction at 5%) for the probability for IMT in cMFC cases stratified by the degree of platelet granularity. The horizontal axis (x-axis) represents time in years, and the vertical axis (y-axis) shows the cumulative proportion of patients that are treated with IMT. The red line indicates the cases with high granularity (≧148.7 units for Cell-Dyn code PIMN) and the blue line indicates the curve for cases with low granularity (<148.7 units). The 95% confidence interval for each curve is also shown. The dotted lines represent the median time to start treatment with IMT in the group with a high and a low PIMN, which were 98 days and 1236 days, respectively. HR, hazard ratio; CI, confidence interval; PIMN, platelet count measured at intermediate angle scatter of 7 degrees; IMT; corticosteroid-sparing immunomodulatory therapy; d, days.

Next, we investigated if blood cell composition parameters could be used as a marker for treatment with systemic corticosteroid-sparing immunomodulatory therapy (IMT). For this analysis we included 69 patients with available blood cell composition data at time of the disease onset (first visit) and without systemic treatment. First, we filtered for parameters that were most associated with early start of IMT by comparing cases that started IMT within the first 6 months *versus* other cases using a likelihood ratio test. This analysis identified 9 parameters (*P_LRT_* <0.05) considered for further investigation, including several parameters related to blood platelets. Cox proportional hazards analysis for these 9 parameters (adjusting for age and sex) identified increased platelet granularity (≧148.7 units, Cell-Dyn code *PIMN*) as a risk factor for treatment with IMT in cMFC patients (hazard ratio = 2.3, 95% confidence interval = 1.28-4.14, *P_adj_* = 0.049) (**Table 4**). Correcting for disease activity (added as a covariate in addition to age and sex in the model) moderately affected the association between platelet granularity and treatment with IMT (*P_adj: age+sex+activity_=* 0.13). The median time between the first presentation in the UMC Utrecht and the start of IMT was 98 days (0.3 years) for cases with increased platelet granularity (≧148.7 units), and 1236 days (3.4 years) in cases without increased granularity (<148.7 units) (**Figure 2**). Note that the platelet granularity was not significantly different between the subtypes of cMFC.

**Table 4:** The results for the cox proportional hazards model for the probability for treatment with IMT in cMFC cases stratified by the degree of platelet granularity.

Description	Cell-Dyn code	Median (IQR)	Cut-off	N (high/low)	HR (95% CI)		*P-*value	*P_adj_-*value
Platelet granularity	*PIMN*	148.4 (144.4-152.6)	148.7	32/36	Univariate	2.19 (1.23-3.90)	0.008	0.038
					Multivariate (age + sex)	2.30 (1.28-4.14)	0.005	0.049
					Multivariate (age + sex + disease activity)	1.98 (1.11-3.56)	0.02	0.13

The results for both the univariate model and the multivariate model including the covariates age and sex are demonstrated both without (P-value) and with (P_adj_-value) false discovery rate correction at 5%.

MT, corticosteroid-sparing immunomodulatory therapy; cMFC, central multifocal choroiditis; IQR, interquartile range; HR, hazard ratio; CI, confidence interval; PIMN, platelet count measured at intermediate angle scatter of 7 degrees.

## Discussion

In this study, we investigated differences in blood cell composition parameters between patients with cMFC and control subjects. Although overall the differences were moderate, we identified a decrease in monocytes linked to disease activity in cMFC. We further revealed that an increased platelet granularity is a risk factor for treatment with IMT.

Although none of our cMFC cases exhibited a monocyte count below 0.2x10^9^/L (i.e., monocytopenia), the number and percentage of blood monocytes were decreased as compared to healthy controls. Monocytes are inflammatory cells central to immune pathology in a wide variety of chronic inflammatory conditions and our data support that these cells are related to disease activity in cMFC (22). Decreased monocyte counts have been reported in chronic inflammatory conditions, such as systemic lupus erythematosus (23), and eye inflammation, including in Fuchs uveitis syndrome (22, 24). Since treatment with systemic corticosteroids tends to influence the monocyte count we adjusted for corticosteroids use (**Table 2**). Adjusting for concomitant corticosteroid use further strengthened the observation of decreased monocytes in cMFC. This makes it tempting to speculate that in the active stage of cMFC, monocytes become activated, and migrate into tissue to differentiate into macrophages as is observed in other forms of vasculitis (25–27). This may possibly result in a relatively low monocyte count. Although a routine hematology analyzer does not differentiate monocyte subsets, the monocyte population is heterogeneous and single cell technology could quickly reveal novel subsets that play a role in ocular inflammation (22, 28). Application of single-cell transcriptomics to leukocytes from cMFC patients will be required to dissect the precise changes in composition of subsets of leukocytes in this condition.

We detected that a subset of patients exhibited enhanced FL3 neutrophil granulocyte fluorescence. Although autofluorescence of neutrophils can drop after bacterial infection (29), an increase in fluorescence of blood neutrophils is only reported after clozapine use (18), which made us consider to evaluate the exposure to fluorescent agents commonly used for ocular imaging. Although our evidence remains circumstantial, fluorescein and indocyanine green angiography within 8 hours prior to sampling was able to explain nearly all cases with enhanced neutrophil FL3 signals. The most simple explanation is that neutrophils take up the fluorescein dye which subsequently is measured in the FL3 channel of the automated hematology analyzer as an increased fluorescence of the neutrophils (30–32).

Patients with cMFC show great heterogeneity in disease course, considering relapse rate and in line with this whether or not they require systemic treatment with IMT (1). Postponing treatment with IMT may result in irreversible retinal damage and deterioration of visual functioning. On the other hand, overtreatment is a burden for the patient and increases the risk for considerable side-effects, which may compromise future therapy compliance (33). Approaches that aid in patient stratification to predict at an early stage whether or not the patient will require systemic treatment with IMT will help alleviate the raised concerns on adequate application of IMT in the treatment of cMFC. The use of IMT in this patient group is also challenging considering that patients are often young and in the fertile stage of life, which warrant accurate predictive tools to help guide shared decision making by uveitis experts and patients. In this study, treatment with IMT is considered a surrogate marker for disease severity since patients with more severe disease are more likely to start treatment with IMT. Though one should keep in mind that the decision to start with treatment with IMT is dependent on more factors such as bilateral ocular involvement, macular involvement, development of choroidal neovascularization and the preference of the patient.

In an attempt to provide a framework for patient risk stratification in the context of IMT, we deliberately used data from a routine hematology analyzer that is widely available and thus may allow prompt adaptation of its results in a clinical setting. We discovered that patients with a relatively high granularity (i.e., cell “complexity”) of platelets were at higher risk for starting IMT with a median time to start with IMT that was approximately 10-fold shorter compared to cases with relatively low granularity. The Cell-Dyn Sapphire hematology analyzer has high sensitivity and fully automatically assesses the whole blood composition using optical light scatter and fluorescent signals, of which the platelet complexity is detected under a 7° angle light scatter (34) which is very similar to SSC from flow cytometer (35). Although not exceeding the threshold for statistical significance in the Cox regression, other platelets parameters were also linked to IMT in cMFC, including the “platelet distribution width” (cases that started IMT within the first 6 months *versus* other cases, *P_LRT_* = 0.01) and “mean polarized platelets” (*P_LRT_* =0.008), suggesting that besides platelets complexity also the size of platelets is linked to the risk for IMT.

The disease mechanisms driving cMFC are poorly understood, but immune pathology is considered central to its pathogenesis. Previous studies have linked variants in complement genes (i.e., *CFH* and *CFB* genes) to susceptibility to cMFC (13). This is of interest, because increasing evidence supports a close relationship between complement and platelets (36). Therefore, platelets are increasingly recognized as key contributors to immune responses and are involved in disease mechanisms of several chronic inflammatory diseases (37–39). This relation is thought to be mediated by the observation that platelets can secrete proinflammatory cytokines and chemokines stored in granules which can activate the complement cascade. Moreover, on the other hand complement activation can also lead to platelet activation (36, 40). Immature platelets are generally larger in size, contain higher dense granules content and are thought to be more hyper-reactive than the mature platelets (41). Possibly, the increased platelet granularity observed in cMFC patients may reflect an increased proportion of immature platelets. This concept is supported by the suggestive association of the increased size of the platelets (parameter *platelet distribution width*) since this is also associated with immaturity of platelets. Interestingly, in the literature a positive correlation was found between the proportion of immature platelets, the size of platelets and the disease activity score in patients with systemic lupus erythematosus (SLE) (42). SLE is an autoimmune disease primarily affecting women, similar to cMFC. Moreover, the inflammatory choriocapillaropathy observed in cMFC is in fact a form of choroidal vasculitis (43). In systemic vasculitis including giant cell arthritis and Behcet disease, markers of platelet activation are observed to be higher during active disease (44). Moreover, Von Willebrand Factor, a marker for endothelial cell activation and a mediator for platelet activation, is observed to be increased in Behcet disease and ischemic retinal vasculitis (45). The data used in this study reports on morphology and enumeration of cells and not on functionality. Therefore we are unable to determine the relationship between platelet activity and the start of treatment with IMT.

It is tempting to speculate that in cMFC, platelets play a role in the pathophysiology of cMFC. We suggest that the association between platelet granularity and the start of IMT we observed in this study could be explained by the fact that both the platelet granularity and the start of IMT are related to the severity of the inflammation in the choriocapillaris. A proposed disease mechanism is that inappropriate complement regulation leads to endothelial activation which on its turn mediates platelet activation. Activated platelets can form platelet aggregates in the choriocapillaris, a phenomenon known to occur in cutaneous small vessel vasculitis (46). Hypothetically, these platelet aggregates form microthrombi in the choriocapillaris resulting in capillary dropout as observed as hypofluorescent areas on indocyanine green angiography imaging. Nevertheless more research is needed, particularly regarding the functionality of platelets, in patients with cMFC to confirm our findings and to further unravel the pathophysiological mechanism of cMFC. Possibly, detailed analysis of platelets function, using analysis of surface receptors can help dissect the platelet subsets affected in cMFC and help improve patient stratification approaches (47). Moreover, is would be interesting to explore the presence of markers of endothelial activation, including Von Willebrand Factor, or antibodies against platelets, for example antiphospholipid antibodies, to attempt to further unravel the pathophysiology of cMFC.

The results of this study should be interpreted with the knowledge of possible limitations of this study. The number of cases we used for this study is based on available data in the UPOD database rather than determined by a power calculation. Moreover, the control subjects used in this study were relatively young individuals that underwent blood sampling in the context of a periodic occupational health examination. These subjects were not checked for comorbidities, though considering the young age it is unlikely, but cannot be ruled out, that these subjects have underlying comorbidities.

To our knowledge, this is the first study reporting on readily available blood cell composition parameters in patients with cMFC. We evaluated these parameters in a large group of patients, especially considering the rarity of the disease. In our study population most patients were diagnosed with MFC and only a minority with the other subtypes PIC, PPM, RPC or SC. It will be interesting to compare changes in platelets in subgroup analysis using larger cohorts in future studies.

In conclusion, we found a decreased level of monocytes in patients compared to control subjects. Moreover, increased platelet granularity could potentially be used as a marker for treatment with IMT.

## Data Availability Statement

The raw data supporting the conclusions of this article will be made available by the authors, without undue reservation.

## Ethics Statement

The studies involving human participants were reviewed and approved by Institutional review board of the University Medical Centre (UMC) of Utrecht. The patients/participants provided their written informed consent to participate in this study.

## Author Contributions

EG: contributed to the design of the study, performed the analyses and wrote the manuscript. JO-vN: contributed to the design of the study, contributed to the discussion of the manuscript. IH: provided the data. SH: contributed to the design of the study, provided the data. JB: contributed to the design of the study, contributed to the discussion of the manuscript. JK: contributed to the design of the study, performed the analyses and wrote the article. All authors contributed to the article and approved the submitted version.

## Funding

Dr. F.P. Fischer Foundation; Achtersloot 212-C, 3401 NZ IJsselstein, the Netherlands. Foundation Beheer het Schild; Wolfhezerweg 101, 6874 AD Wolfheze, the Netherlands. Landelijke Stichting voor Blinden en Slechtzienden (LSBS); Galvanistraat 1, 6716 AE Ede, the Netherlands; Rotterdamse Stichting Blindenbelangen (RBS); Schiekade 77, 3033 BE Rotterdam, the Netherlands; Stichting Louise Rottinghuis Fonds; Ringenum 6, 9934 PM Delfzijl, the Netherlands. Oogfonds; Churchilllaan 11, 3527 GV Utrecht, the Netherlands.

## Conflict of Interest

SH was supported by a fellowship by Abbott Diagnostics and IH declared that Abbott Hematology and Abbott Diagnostics have contracts with the Central Diagnostic Laboratory.

The remaining authors declare that the research was conducted in the absence of any commercial or financial relationships that could be construed as a potential conflict of interest.

## Publisher’s Note

All claims expressed in this article are solely those of the authors and do not necessarily represent those of their affiliated organizations, or those of the publisher, the editors and the reviewers. Any product that may be evaluated in this article, or claim that may be made by its manufacturer, is not guaranteed or endorsed by the publisher.
