# Supplementary material for: Central Multifocal Choroiditis: Platelet Granularity as a Potential Marker for Treatment With Steroid-Sparing Immunomodulatory Therapy
Source: Front Ophthalmol (Lausanne). 2021 Nov 25;1:784848. doi: 10.3389/fopht.2021.784848 (PMC11182307; doi:10.3389/fopht.2021.784848)
Supplement: Supplementary file 1 [file Table_1.docx]

**Supplementary Table S1.** Cell-Dyn Codes blood cell composition parameters measured within the UPOD database

| *Leukocytes* | | | | |
| --- | --- | --- | --- | --- |
| WBC | White blood cell count (x 10^9^/L) | WVF | White blood cell viability fraction | |
| *Neutrophil granulocytes* | | | | |
| NEU | Neutrophil count (x 10^9^/L) | PNEU | % neutrophils of leukocyte count | |
| SEG | Segmented neutrophil count (x 10^9^/L) | PSEG | % segmented neutrophils of leukocyte count | |
| BND | Banded neutrophil count (x 10^9^/L) | PBND | % banded neutrophils of leukocyte count | |
| IG | Immature granulocyte count (x 10^9^/L) | PIG | % immature granulocytes of leukocyte count | |
| NAMN | Mean neutrophil 0 degrees (ALL) | NACV | CV% neutrophil 0 degrees (ALL) | |
| NIMN | Mean neutrophil 7 degrees (IAS) | NICV | CV% neutrophil 7 degrees (IAS) | |
| NPMN | Mean neutrophil 90 degrees polarized (PSS) | NPCV | CV% neutrophil 90 degrees polarized (PSS) | |
| NDMN | Mean neutrophil 90 degrees depolarized (DSS) | NDCV | CV% neutrophil 90 degrees depolarized (DSS) | |
| NFMN | Mean fluorescence neutrophil (FL3) | NFCV | CV% fluorescence neutrophil (FL3) | |
| *Lymphocytes* | | | | |
| LYM | Lymphocyte count (x 10^9^/L) | PLYM | % lymphocytes of leukocyte count | |
| LYME | Lymphocyte count without atypical lymphocytes (x 10^9^/L) | PLYME | % lymphocytes without atypical lymphocytes of leukocyte count | |
| VLYM | Atypical lymphocyte count (x 10^9^/L) | PVLYM | % atypical lymphocytes of leukocyte count | |
| LAMN | Mean lymphocyte 0 degrees (ALL) | LACV | CV% lymphocyte 0 degrees (ALL) | |
| LIMN | Mean lymphocyte 7 degrees (IAS) | LICV | CV% lymphocyte 7 degrees (IAS) | |
| *Monocytes* | | | | |
| MON | Monocyte count (x 10^9^/L) | PMON | % monocytes of leukocyte count | |
| MONE | Monocyte count without blasts (x 10^9^/L) | PMONE | % monocytes without blasts of leukocyte count | |
| BLST | Blast count without monocytes (x 10^9^/L) | PBLST | % blasts without monocytes of leukocyte count | |
| *Eosinophils + Basophils* | | | | |
| EOS | Eosinophil count (x 10^9^/L) | PEOS | % eosinophils of leukocyte count | |
| BAS | Basophil count (x 10^9^/L) | PBAS | % basophils of leukocyte count | |
| *Platelets* | | | | |
| PLT | Platelet count (x 10^9^/L) | PCT | Platelet crit (ml/L) | |
| PLTO | Platelet count (x 10^9^/L) optical measurement | PLTI | Platelet count (x 10^9^/L) impedance measurement | |
| PDW | Platelet distribution width | prP | Enumeration of reticulated platelets | |
| PIMN | Mean platelet 7 degrees (IAS) | PICV | CV% platelet 7 degrees (IAS) | |
| PPMN | Mean platelet 90 degrees polarized (PSS) | PPCV | CV% platelet 90 degrees polarized (PSS) | |
| MPV | Mean platelet volume (fL) |  |  | |
| *Erythrocytes* | | | | |
| RBCO | Rbc count (x 10^9^/L) optical measurement | RBCI | | Rbc count (x 10^9^/L) impedance measurement |
| RBCIMN | Mean rbc 7 degrees (IAS) | RBCICV | | CV% rbc 7 degrees (IAS) |
| RBCFMN | Mean fluorescence rbc (FL3) | RBCFCV | | CV% fluorescence rbc (FL3) |
| MCV | Mean corpuscular volume rbc fL | MCH | | Mean corpuscular hemoglobin rbc fmol |
| MCHC | Mean corpuscular hemoglobin concentration rbc mmol/L | MCH USA | | Mean corpuscular hemoglobin rbc (USA unit) |
| MCHC USA | Mean corpuscular hemoglobin concentration rbc (USA unit) | RDW | | Red blood cell distribution width |
| PMIC | % rbc with volume of less than 60 fL | PMAC | | % rbc with volume greater than 120 fL |
| HB | Hemoglobin (mmol/L) | HB USA | | Hemoglobin USA units |
| HT | Hematocrit (L/L) | PHPO | | % rbc with a hemoglobin concentration < 28 g/dL |
| PHPR | % rbc with a hemoglobin concentration > 41 g/dL | HDW | | CV% hemoglobin concentration |
| RETC | Reticulocyte count (x 10^9^/L) | PRETC | | % reticulocytes of rbc count |
| MCVR | Mean corpuscular volume reticulocyte fL | MCHR | | Mean corpuscular hemoglobin reticulocyte fmol |
| MCHCR | Mean corpuscular hemoglobin concentration reticulocyte mmol/L | IRF | | Immature reticulocyte fraction |
| RTCFMN | Mean fluorescence reticulocyte (FL3) | RTCFCV | | CV% fluorescence reticulocyte (FL3) |
| NRBC | Erythroblast count (x 10^9^/L) | PNRBC | | % erythroblasts per 100 white blood cells |
